# Potential External Contamination with Bisphenol A and Other Ubiquitous Organic Environmental Chemicals during Biomonitoring Analysis: An Elusive Laboratory Challenge

**DOI:** 10.1289/ehp.1206093

**Published:** 2013-01-16

**Authors:** Xiaoyun Ye, Xiaoliu Zhou, Ryan Hennings, Joshua Kramer, Antonia M. Calafat

**Affiliations:** Division of Laboratory Sciences, National Center for Environmental Health, Centers for Disease Control and Prevention, Atlanta, Georgia, USA

**Keywords:** benzophenone-3, biomonitoring, bisphenol A, exposure assessment, parabens, reagent blank, triclosan

## Abstract

Background: Biomonitoring studies are conducted to assess internal dose (i.e., body burden) to environmental chemicals. However, because of the ubiquitous presence in the environment of some of these chemicals, such as bisphenol A (BPA), external contamination during handling and analysis of the biospecimens collected for biomonitoring evaluations could compromise the reported concentrations of such chemicals.

Objectives: We examined the contamination with the target analytes during analysis of biological specimens in biomonitoring laboratories equipped with state-of-the-art analytical instrumentation.

Discussions: We present several case studies using the quantitative determination of BPA and other organic chemicals (i.e., benzophenone-3, triclosan, parabens) in human urine, milk, and serum to identify potential contamination sources when the biomarkers measured are ubiquitous environmental contaminants.

Conclusions: Contamination with target analytes during biomonitoring analysis could result from solvents and reagents, the experimental apparatus used, the laboratory environment, and/or even the analyst. For biomonotoring data to be valid—even when obtained from high-quality analytical methods and good laboratory practices—the following practices must be followed to identify and track unintended contamination with the target analytes during analysis of the biological specimens: strict quality control measures including use of laboratory blanks; replicate analyses; engineering controls (e.g., clean rooms, biosafety cabinets) as needed; and homogeneous matrix-based quality control materials within the expected concentration ranges of the study samples.

Humans are exposed to environmental chemicals through industrial and indoor air pollution, diet, and use of personal care and consumer products. Biomonitoring (i.e., measurement of the environmental chemicals or their metabolites in biological speciments) is widely used to assess human internal exposure (i.e., body burden) to these chemicals [Centers for Disease Control and Prevention (CDC) 2012; Den Hond et al. 2011; Frery et al. 2012; Health Canada 2010; Kim et al. 2011; National Research Council 2006; Suzuki et al. 2010].

Proper biomonitoring practices take into account the selection of the relevant biomarker and biomonitoring matrix, the potential impact of the collection protocol on the biomarker levels in the sample, as well as the integrity of the sample during its collection, handling, storage, and analysis (Calafat and Needham 2009). Furthermore, accurate and precise highly sensitive and selective multianalyte analytical methods for extraction, separation, and detection of the environmental chemicals are required to obtain valid biomonitoring data (Angerer et al. 2007). Participation in external quality assessment programs [e.g., Arctic Monitoring and Assessment Program (Institut National de Santé Publique du Québec 2012), German External Quality Assessment Scheme (G-EQUAS; Social and Environmental Medicine of the University Erlangen-Nuremberg 2012)] and the use of standard reference materials (SRMs) from the National Institute of Standards and Technology (Keller et al. 2010; Schantz et al. 2013) are very useful tools to evaluate method accuracy.

However, even with the application of sophisticated and accurate methods, external contamination with some ubiquitous environmental organic chemicals, such as bisphenol A (BPA), polybrominated diphenyl ethers (PBDEs), and polychlorinated biphenyls (PCBs), during sample analysis can compromise the analytical determination of these compounds [Alcock et al. 1994; Sjödin et al. 2004; World Health Organization (WHO) 2011]. External contamination can even preclude accurate analyses of phthalate diesters, which are detected in the cleanest laboratory reagents, sampling equipment, and analytical apparatus. Therefore, assessing human exposure to phthalates is routinely done by measuring biomarkers that cannot be formed in the environment (e.g., oxidized metabolites of phthalates) instead of the phthalate diesters themselves (Koch and Calafat 2009).

Field blanks have been used to assess potential contamination during collection, processing, and/or transport of environmental samples (National Institute for Occupational Safety and Health 1994) and the importance of using field blanks in biomonitoring studies has been presented (National Research Council 2006; WHO 2011). Furthermore, it is well recognized that reagent or quality control blank (QCB) and quality control (QC) samples, when used and evaluated properly, are invaluable to monitoring potential contamination during analysis and the accuracy/precision of the measurements (Taylor 1987).

Several reports have investigated potential sources of contamination with ubiquitous organic chemicals during sample analysis and how to mimimize contamination in a typical biomonitoring laboratory setup (Alcock et al. 1994; Markham et al. 2010; Sjödin et al. 2004; Xie et al. 2006). Here we present several case studies using the measurement of BPA and other ubiquitous environmental organic chemicals (e.g., benzophenone-3, triclosan, parabens) in human urine, milk, and serum by on-line solid phase extraction coupled to isotope dilution–high performance liquid chromatography–tandem mass spectrometry (on-line SPE-HPLC-MS/MS) to discuss potential contamination scenarios during analysis, including the reagents and apparatus used, the laboratory environment, and the analyst.

## Discussion

*Contamination from solvents and reagents*. During analysis, contamination from solvents (including water) or reagents is monitored through the calculated concentration and the signal/noise (S/N) ratio of the reagent blank or QCB peak (Taylor 1987). However, the QCB calculated concentration per se might not reveal systematic contamination during analysis (e.g., as when the same reagents/solvents are used to prepare both QCB and standards). Concentrations of samples, including the QCB, are calculated from a calibration curve constructed by plotting the instrument response of analytical standards versus their known concentrations after automatically subtracting the *y*-intercept—the instrument response at a zero concentration—from the response of the samples (Taylor 1987). If the *y*-intercept includes a contribution from the contamination of reagents/solvents present in both the analytical standards and the QCB, the calculated concentration of the QCB will be around zero or below the limit of detection (LOD) of the method. Therefore, reagent or solvent contamination can not be revealed by the calculated QCB concentration, but only through the higher than expected S/N ratio of the QCB (i.e., S/N > 3).

**Figure 1:**
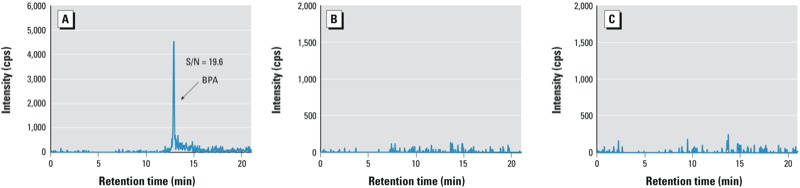
HPLC-MS/MS–extracted ion chromatograms of BPA (m/z ion transition 227/133) of QCBs using a method for the analysis of urine. (*A*) QCB with BPA interference in the water used in the SPE and HPLC mobile phases (QCB signal is equivalent to a concentration of 1.5 µg/L); (*B*) QCB without BPA interference; (*C*) QCB with BPA interference in the mobile phase, but adding guard cartridges after SPE and HPLC pumps.

To measure BPA in urine, we have used methanol and water as mobile phases of an on-line SPE-HPLC-MS/MS system (Ye et al. 2005, 2006). In one instance, right after the laboratory water purification system had been serviced during its regular preventative maintenance, we observed a high S/N ratio (19.6) of the BPA peak for the QCBs (Figure 1A). However, the calculated BPA concentration of the QCB was below the LOD, suggesting that we had a systematic contamination. Further investigation confirmed that the contamination was in the water and resulted from BPA leaching from a polyethersulfone filter installed during the preventative maintenance of the water purification system. After replacing this filter with another brand of polyethersulfone filter, the QCB S/N ratio went back to its normal levels (Figure 1B), suggesting that the latter filter, albeit made from the same material as the first, did not leach BPA.

**Figure 2:**
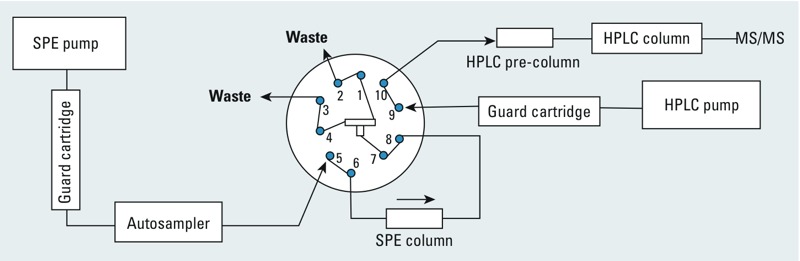
On-line SPE-HPLC-MS/MS configuration, adapted from a previous publication (Ye et al. 2008), with guard cartridges after SPE and HPLC pumps. The 10-port switching valve configuration shown corresponds to the time period when the sample is loaded onto the SPE cartridge.

To minimize the impact of low-level contamination of HPLC and SPE mobile phases with the target compounds (in this case, BPA), we modified our experimental configuration by adding guard cartridges to both mobile phase lines, similar to the approach we used before for filtering out an interference of perfluorooctanoate (Kuklenyik et al. 2009). Specifically, we added two C18 guard cartridges, one right after the SPE pump and another right after the HPLC pump (Figure 2). The SPE guard cartridge retains BPA during the loading of the sample on the SPE column with a relatively low organic content [e.g., 20% methanol in water (Ye et al. 2005)], so the interference of BPA from the SPE mobile phase will not be loaded onto the SPE column. The BPA interference trapped in the SPE guard cartridge is flushed to waste during the SPE column regeneration step. When the BPA contaminant from the HPLC mobile phase retained on the HPLC guard cartridge elutes onto the HPLC analytical column with the HPLC gradient, the additional length of the guard cartridge delays the elution of the BPA contaminant compared with that of the “true” BPA peak from standards, QCs, and study samples. Figure 1C shows the extracted ion chromatograms of BPA from QCBs with BPA interference obtained when using contaminated water in both SPE and HPLC mobile phases, but adding guard cartridges after the SPE and HPLC pumps. Compared with Figure 1A, the BPA contamination from the SPE solvent is completely removed. Noteworthy, the absence of another BPA peak at the expected longer retention time suggests that the BPA interference from the water in the HPLC mobile phase is negligible compared with the BPA in the water of the SPE mobile phase, an interference that had been preconcentrated on the SPE cartridge.

When contamination affecting standards, QC materials, and study samples cannot be avoided completely, the concentration of study samples is adjusted by subtracting the contribution from the QCB. However, a high QCB contribution will increase the variability of measurements at low-concentration ranges. In the example above, contamination with BPA of the water purification system increased the frequency of out of control values of the low-concentration QC materials (~ 2.5 µg/L), which, unfortunately, is also around the median urinary concentration of BPA for the general population in various countries (CDC 2012; Health Canada 2010; Koch et al. 2012). Together, these findings show that careful evaluation of the concentration and S/N ratio patterns of the QCBs, as well as having homogeneous matrix-based QC materials at the relevant concentrations (e.g., general population median), were instrumental in identifying the contamination of BPA from the deionized water.

**Figure 3:**
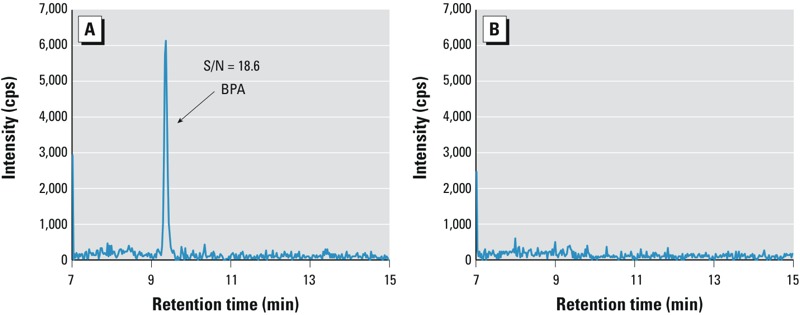
HPLC-MS/MS extracted ion chromatograms of BPA (m/z ion transition 227/133) of QCBs using a method for the analysis of milk. (*A*) QCB prepared in a plastic microcentrifuge tube; (*B*) QCB prepared in conical silanized glass autosampler vials.

*Contamination of experimental apparatus.* The experimental apparatus (e.g., septum-equipped test tubes) could be a contamination source when measuring PBDEs (Sjödin et al. 2004). Similarly, because BPA can leach from plastic [National Toxicology Program (NTP) 2008], contamination of BPA from plasticware is also possible. On one occasion, while analyzing breast milk for BPA by on-line SPE-HPLC-MS/MS (Ye et al. 2008), we observed not only a high S/N ratio (S/N = 18.6) of the QCB signal, but also that its calculated BPA concentration was consistently 1–2 µg/L above the LOD of 0.3 µg/L (Figure 3A). These combined results indicated that the contamination of BPA was not systematic (i.e., at least not in the standards). The sample pretreatment included precipitation of milk proteins by methanol, followed by centrifugation of the milk in a disposable microcentrifuge plastic tube. We added the same solvents to standards, QCBs, and milk samples, but prepared the solvent-based standards directly in silanized autosampler glass vials without the centrifugation step. We confirmed that the BPA contamination in the QCBs came from the leaching of BPA from a specific lot of microcentrifuge tubes, even though the tubes were presented as made of polypropylene, which is not known to contain BPA. When we prepared the QCBs in silanized glass autosampler vials, the BPA contamination dissapeared (Figure 3B). To avoid future contamination, we modified our procedure and prepared the milk samples in conical silanized glass vials instead of plastic microcentrifuge tubes; we also reduced the spin speed to avoid breaking the glass vials during centrifugation. Our findings above reiterate the importance not only of including QCBs in each analytical batch, but that the QCBs have to go through the exact same processing protocols, including using the same apparatus, as the study samples.

*Contamination in the laboratory environment*. Indoor air is a known source of PCBs, particularly the low molecular weight congeners (Alcock et al. 1994). Similarly, because BPA, triclosan, and parabens have been detected in indoor dust (Fan et al. 2010; Geens et al. 2009; Liao et al. 2012), contamination with these ubiquitous chemicals could also come from the laboratory environment. In another situation, we measured the concentrations of free and total (free + conjugated) BPA in serum as part of an exposure study. We followed strict QC protocols, namely having at least two QCBs, two low-concentration QCs, and two high-concentration QCs in each analytical batch with 50 study samples, and including replicate analysis of at least 10% of study samples. Even with these cautionary measures, we observed random contamination of BPA in about 2% of the replicate analyses (Teeguarden et al. 2011). We first ruled out the potential contamination with BPA from solvents and reagents. Because BPA can be used in the manufacture of certain plastics (NTP 2008) and can be present in paper products (Geens et al. 2012; Liao and Kannan 2011), we systematically evaluated the experimental apparatus used and the materials present in our laboratory. We identified the absorbent underpad used to cover the workspace in the laboratory benches as the source of the contamination. We speculate that the random contamination originated when underpad fibers got into the autosampler vials during preparation of the serum for analysis. We eliminated this contamination after we replaced the absorbent underpad with other bench top protection sheets that did not contain BPA.

In a similar situation, we detected random contamination of triclosan in the QCBs and QCs while analyzing urine (Ye et al. 2005). After ruling out contamination from reagents, solvents, and experimental apparatus, we discovered that shortly before we noticed the contamination, the hand soap in the restrooms dispensers had been replaced with hand soap containing triclosan, a broad-spectrum antimicrobial agent widely used in soaps in the U.S. market (Perencevich et al. 2001). Despite using proper personal protection equipment for sample preparation in the laboratory, including changing gloves often as needed during sample preparation, the analysts, after washing their hands with the antibacterial soap, randomly contaminated some of the samples. The triclosan contamination disappeared after replacing triclosan-containing hand soap in the restroom dispensers with triclosan-free soap.

These findings confirm that the general laboratory environment and its immediate surroundings can also be a potential contamination source. Noteworthy, because of its random pattern, this contamination is usually difficult to identify and track down even by experienced researchers adhering to a comprehensive QC protocol. One approach for identifying the potential contamination from laboratory environment involves running replicate analysis, particularly when the number of study samples is small. If replicate analysis is not possible (e.g, limited sample volume), another alternative is including additional QCBs and QCs within the analytical batch.

The analyst can also be the contamination source, especially for chemicals present in consumer or personal care products. In another example, we noticed random, yet often repeated contamination with triclosan in some QCBs and QCs included in the batches prepared by one analyst, but not others. After ruling out the potential contamination sources discussed above and confirming that this person followed the required standard operating procedures, we inventoried all of the situations, both in and outside the workplace, where the analyst could come in contact with triclosan-containing products (Department of Health and Human Services 2007; Perencevich et al. 2001). At the end of an investigation that took several weeks, we determined that the contamination started after the analyst changed the toothpaste at home to one that contained triclosan. In another instance, we encountered a similar random contamination with benzophenone-3 and parabens. In this case, the contamination sources were the analyst’s sunscreen lotion and underarm deodorant which contained benzophenone-3 and parabens (Department of Health and Human Services 2007). In both cases, we realized the occurence of contamination through the abnormal results of the QCBs and QCs. After we identified the sources of contamination, and although the analysts did not have to change their habits related to personal care products use, we managed to reduce the potential contamination to negligible levels by conducting all sample preparation procedures in a biological safety cabinet.

## Conclusions

Unintended contamination with ubiquitous environmental chemicals, such as BPA and other organic compounds (e.g., benzophenone-3, triclosan, parabens), during biomonitoring analyses is possible, even with state-of-the-art analytical methods and laboratory facilities. Unfortunately, until all of the environmental sources of these chemicals are known, totally eliminating external contamination is practically impossible. However, judicious application of the measures below will allow the identification of contamination scenarios, thus facilitating the implementation of measures to isolate and track external contamination and minimize as much as possible its recurrence and impact.

Evaluate the QCB (i.e., reagent blank) by checking both the calculated concentration and the S/N ratio of the QCB peak to assess any systematic or non-systematic contamination.
Prepare the QCBs using the same procedure and apparatus as the unknown study samples to avoid missing labware and apparatus potential contamination.
Use guard columns to filter out potential interference contaminants from the SPE mobile phases or separate the chromatograhic peak of the target analyte in the study samples from its interference peak in the HPLC mobile phases.
Conduct replicate analyses (e.g., 5%) of study samples to identify random contamination from the laboratory environment and/or analyst, especially when the number of study samples is small. When limited sample volume precludes replicate analyses, include additional blanks randomly placed within the analytical batch.
Prepare the samples for analysis in a controlled environment (i.e., biological safety cabinet, clean room) if contamination from the analyst or the environment (e.g., air, dust) is suspected.
Use homogeneous matrix-matched QC materials at concentrations within the expected concentration ranges of the study samples.
When possible, participate in external quality assessment programs or use SRMs to evaluate the accuracy of the measurement. Unfortunately, for nonpersistent environmental organic chemicals (e.g., BPA, benzophenone-3, triclosan, parabens) commercially available SRMs do not exist and only BPA is routinely included in G-EQUAS. Expanding such quality assessment programs and characterizing SRMs to include these chemicals are critical to improve the accuracy of biomonitoring methods.

In summary, valid biomonotoring data require integrating numerous preanalytical and analytical steps. First, adequate selection of the most relevant biomarkers and biomonotoring matrices as well as collection, handling, shipping, and storage procedures to preserve the integrity of the specimen and the target analytes are needed before starting sample analyses. Second, use of sensitive, selective, and accurate analytical methods and state-of-the-art laboratory facilities. Third, use of highly trained laboratory personnel not only to operate sophisticated instrumentation, but also to recognize situations that may compromise both the integrity of the biological specimen and the validity of its analysis. Last, use of good laboratory practices and implementation of measures as discussed above to minimize unintended contamination of the biological specimens with the target analytes during analysis. We strongly believe that biomonitoring studies that maximize the harmonization of the various disciplines and expertises (e.g., field investigation, laboratory analysis) will considerably strengthen the exposure assessment.
